# There Is no Theory-Free Measure of “Swaps” in Visual Working Memory Experiments

**DOI:** 10.1007/s42113-022-00150-5

**Published:** 2022-09-14

**Authors:** Jamal R. Williams, Maria M. Robinson, Timothy F. Brady

**Affiliations:** 1 Department of Psychology, University of California San Diego, 9500 Gilman Dr. #0109, La Jolla, CA 92093, USA

**Keywords:** Visual working memory, Swap, Model-based measurement

## Abstract

Visual working memory is highly limited, and its capacity is tied to many indices of cognitive function. For this reason, there is much interest in understanding its architecture and the sources of its limited capacity. As part of this research effort, researchers often attempt to decompose visual working memory errors into different kinds of errors, with different origins. One of the most common kinds of memory error is referred to as a “swap,” where people report a value that closely resembles an item that was not probed (e.g., an incorrect, non-target item). This is typically assumed to reflect confusions, like location binding errors, which result in the wrong item being reported. Capturing swap rates reliably and validly is of great importance because it permits researchers to accurately decompose different sources of memory errors and elucidate the processes that give rise to them. Here, we ask whether different visual working memory models yield robust and consistent estimates of swap rates. This is a major gap in the literature because in both empirical and modeling work, researchers measure swaps without motivating their choice of swap model. Therefore, we use extensive parameter recovery simulations with three mainstream swap models to demonstrate how the choice of measurement model can result in very large differences in estimated swap rates. We find that these choices can have major implications for how swap rates are estimated to change across conditions. In particular, each of the three models we consider can lead to differential quantitative and qualitative interpretations of the data. Our work serves as a cautionary note to researchers as well as a guide for model-based measurement of visual working memory processes.

The architecture and capacity limit of working memory is of broad interest (Cowan, 2001; Miyake & Shah, 1999), both for its own sake and because it is related to fluid intelligence, reading comprehension, and academic achievement (Alloway & Alloway, 2010; Daneman & Carpenter, 1980; Fukuda et al., 2010). This memory system is extremely limited. For example, in visual working memory, participants struggle to retain accurate information about even 3–4 visual objects for just a few seconds (Luck & Vogel, 1997; Ma et al., 2014; Schurgin et al., 2020).

Assessments of visual working memory capacity and performance over the past decade have commonly relied on continuous reproduction tasks (Wilken & Ma, 2004; Zhang & Luck, 2008). For example, participants might be asked to use a color wheel to reproduce the color that was briefly shown at a particular location in the memory array after a delay (Fig. 1A). In general, when this task is made sufficiently difficult—for example, by using short encoding times, or long delays, or asking people to remember many items at once—participants make many “large” errors, meaning that they report feature values (colors) that deviate substantially away from the true color of the target item. There is great theoretical interest in breaking down the pattern of such “large” errors into separable components, because elucidating the source of these errors has direct implications for the architecture of visual working memory (e.g., Bays et al., 2009; Zhang & Luck, 2008). In particular, work by Zhang and Luck (2008) influentially proposed a mixture model according to which such large errors reflect “pure guessing,” an assumption that broadly entails strict item limits in visual working memory. Much follow-up work has either challenged or built on that view, postulating alternative mechanisms and processes to explain these large errors (e.g., Schurgin et al., 2020; Taylor & Bays, 2020; van den Berg et al., 2012). In the current work, we focus on one such line of research, which is commonly characterized as the decomposition of memory errors into “target-related” vs. “swap” components (Bays et al., 2009; Bays, 2016; Williams et al., 2022).

“Swaps” are the idea that people might make memory confusions and spuriously report on a non-target item that was shown in the memory array but was not probed at test. This can result in large errors, even though it is not a result of a pure guessing process. In the literature on continuous reproduction in visual working memory, Bays et al. (2009) initially characterized such swaps, and proposed an expanded mixture model to measure their prevalence, allowing for both “pure guesses” and “swaps” to explain large errors, beyond the “pure guessing” proposed by Zhang and Luck (2008) to underlie such errors. Bays et al. (2009) found such swap errors are common in such data under particular sets of circumstances (e.g., short encoding times with many items).

Such swap errors may reflect a failure of binding (as binding is known to be difficult in visual working memory tasks, e.g., Wheeler & Treisman, 2002), or simply stochastic noise in the cue dimension causing confusions between items (e.g., error in location, in the task shown in Fig. 1; McMaster et al., 2022; Oberauer & Lin, 2017; Schneegans & Bays, 2017). A systematic study of swap errors is of broad relevance to visual memory researchers because swap errors are conceptually aligned with different theories of visual memory architecture (e.g., different roles for interference, Oberauer & Lin, 2017). Furthermore, evidence suggests that rates of swap errors and other similar “binding failures” are related to both age-related decline and clinical deficits independent of item memory strength (e.g., Peich et al., 2013; Pertzov et al., 2013; Zokaei et al., 2020). Finally, swap models—that is, visual working memory models that quantify swap rates—have been used extensively in empirical and computational modeling work that aims to develop more ecological theories and models of visual working memory, in particular, work that seeks to go beyond the untenable simplifying assumption that visual working memory representations are solely stored independently for each item, instead examining how interactions between memory representations elicit robust memory biases (e.g., Brady & Alvarez, 2015). In the context of such work, swap models are often used to quantify swap rates, with the goal of formally separating swaps (i.e., memory confusions) from other memory biases, like attraction and repulsion between items (e.g., Brady & Alvarez, 2015; Chunharas et al., 2019; Golomb, 2015; Lively et al., 2021).

However, much of this work using swap models does not acknowledge the fact that all extant swap models hinge on strong theoretical and/or parametric assumptions that can have a dramatic influence on swap rate estimates, and accordingly, researchers’ theoretical conclusions. This is partly because the Bays et al. (2009) model has become something of a default swap model in this literature. Indeed, despite its assumptions of the entire long tail of errors arising from a “guessing” component which is generally in conflict with most modern models of visual working memory errors (e.g., Schurgin et al., 2020; Schneegans et al. 2020; Bays, 2015; van den Berg et al., 2012), it is often taken as the definitive measurement model for how often swaps occur (e.g., Chunharas et al., 2019; Emrich et al., 2013; Golomb, 2015; Honig et al., 2020; Lively et al., 2021; McDougle et al., 2019; Peich et al., 2013; Pertzov et al., 2013; Scotti, Hong, Golomb, & Lever, 2021; Shin et al., 2017; Sun et al., 2017; Zokaei et al., 2020). This is done even by those who explicitly do not subscribe to the underlying claim that there are item limits in memory and reject a “pure guessing” processes in visual memory reproduction tasks (e.g., Chunharas, et al., 2019; Lively et al., 2021; Honig et al., 2020; Schneegans, Harrison & Bays, 2021; Shin et al., 2017). This use of the Bays et al. (2009) model arises in part because thus far there has not been a comprehensive overview of extant swap models that systematically examines how different swap models compare, and how violations of these models’ assumptions influence swap rate estimates.

The current paper aims to fill this gap by exploring the assumptions behind several distinct models of “swaps” used in the visual working memory literature, and the extent to which they are consistent with modern takes on visual memory error distributions. In particular, we ask how robust the Bays et al. (2009) model is to different assumptions, like the idea proposed by most modern theories of visual working memory that large errors in reproduction arise, not from a separate pure guessing state but from other kinds of item-based stochasticity (e.g., Bays, 2015; Schurgin et al., 2020; Schneegans et al. 2020; van den Berg et al., 2012), and that items are not stored independently in visual working memory but tend to both attract and repel each other (e.g., Chunharas et al., 2019; Lively et al., 2021; Golomb, 2015; Scotti, Hong, Leber & Golomb, 2021; Bae & Luck, 2017; Chen et al., 2019; Brady & Alvarez, 2011, 2015; Oberauer & Lin, 2017).

We consider 3 prominent models that have been used to measure swap errors: the 3 component model of Bays et al. (2009), as described above (referred to as “Mixture-Swap” from this point forward); the non-parametric swap model of Bays (2016; “NP-Swap”); and the TCC-Swap model proposed by Williams et al. (2022) based on the target confusability competition (TCC) model of Schurgin et al. (2020). We focus on these models because they hinge on qualitatively different theoretical and parametric assumptions and thus examining this set of models provides insight into how a violation of some of these assumptions affects swap rate estimates. Other swap models—for example, a model based on the variable precision theory of van den Berg et al. (2012), or the neural noise theory of Bays (2014)—exist, but would be expected to be qualitatively similar to the TCC-Swap model (Williams et al., 2022) because these models also assume that item-based responses, rather than pure guesses, underlie the long tail of large errors in reproduction tasks. Thus, this set of 3 models naturally captures a reasonable space of possibilities: a model that assumes item-based responses, including swaps, only ever result in small errors, and large errors are pure guesses (Bays et al., 2009); a model that assumes all responses are item-based, including large errors (Williams et al., 2022); and a model that simply seeks to sort the most likely item that originated a response in a non-parametric way without any direct underlying theory of memory responses (Bays, 2016), but which, as a result, implicitly assumes that swap responses are identical in functional form to non-swap responses and thus, like the TCC-Swap model, counts the long tail as item-based.

Why might we expect these models to differ? The core difference between including the long tail in your estimate of swaps (as in Williams et al., 2022 or Bays, 2016) or excluding it and attributing it to guessing (as in Bays et al., 2009) is illustrated in Fig. 2, which shows the exact same simulated error distribution fit on top by TCC-Swap, and on bottom by Mixture-Swap models. In particular, this figure demonstrates that under conditions in which some proportion of responses are centered on the non-target, the TCC-Swap model presumes that many of the responses in the “long tail” are also swap responses; by contrast, the Mixture-Swap model presumes that only the small “bump” above the estimated “guesses” at the non-target location is a result of swap errors. Thus, even for identical distributions of errors, both well fit by the models, the TCC and Bays et al. (2009) models yield radically different swap rate estimates (20% versus 9%, respectively).

The same issue of whether to classify parts of the long tail of errors as swaps or not is also by necessity present in any “non-parametric” swap model: that is, even non-parametric models must make a choice about whether the long tail reflects guesses or item-based responses in order to decide what is a swap (see Fig. 1C–D); and whether it is explicitly stated or not, this choice is inevitably linked with strong theoretical implications. The NP-Swap method assumes, like the TCC-Swap model, that the long tail also contains swaps (since it assumes the same error distribution for swaps and non-swaps). By contrast, less formal non-parametric methods, like manually counting how many responses are closer to a non-target than the target (as in McMaster et al., 2022), only partially count the “long tail” as part of the swap rate (that is, they do not count the full contribution of swaps to the “tails” but also do not fully discount “guess” responses that happen to land near the non-target, as the Bays et al., 2009 model does).

In the remainder of this work, we expand on this basic intuition and use simulations to examine in depth how swap rate estimates of each model are affected under a range of processing assumptions, which are based on contemporary models and views of visual memory processes. In particular, we compare the TCC-Swap (Williams et al., 2022), Bays et al. (2009) swap, and the NP-Swap of Bays (2016) across a variety of memory conditions to elucidate when these models yield roughly similar estimates of swap rates, and when they diverge. It could be that researchers are justified in treating these models as simple measurement models for swaps, independent of their other assumptions, if these models yield swap estimates that are aligned with one another and are, therefore, likely to be robust across different processing assumptions. In contrast, if these models yield substantially different swap rate estimates under reasonable conditions, then researchers are not justified in treating these swap models as general measurement models and should explicitly motivate their choice of model.

We focus our analysis on models that quantify swaps rather than models that seek to explain the causes of swaps (e.g., Oberauer & Lin, 2017; Schneegans & Bays, 2017) because the former are commonly treated as measurement models in the literature, sometimes without a deep consideration of their theoretical assumptions.

Overall, we find dramatic quantitative and qualitative differences in how each model captures swap rates across different processing assumptions. To preview, we find that the Mixture-Swap model and more modern swap models (Bays, 2016; Williams et al., 2022) strongly diverge in their estimate of swap rates when memory is “weak,” but not when memory is “strong.” This occurs because of a fundamental disagreement about whether the “long tail” of errors is viewed as item-based responses (responses due to item confusions) or viewed as pure guesses (Fig. 2). This will result in a major difference in how swap rates are perceived to change across set size and other encoding manipulations. We find that the NP-Swap model of Bays (2016) and TCC model of Williams et al. (2022) largely converge when the necessary preconditions of the NP-Swap model are met. However, the NP-Swap model is strongly dependent on its assumption of complete independence between items—which we believe is frequently violated in real data. Violations of these assumptions yield unreasonable—and even negative—estimates of swap prevalence from the NP-Swap model. We conclude that you cannot measure swaps in a “theory-free” way, meaning that researchers cannot simply treat extant swap models as measurement models that do not carry heavy theoretical baggage, even non-parametric models. Whether the long tail of errors belongs to item-based responses (Bays, 2015; Schurgin et al., 2020; Schneegans et al. 2020; van den Berg et al., 2012) or arises from random, informationless guessing (Zhang & Luck, 2008) is critical to understanding how swap rate changes with memory strength. Furthermore, even the NP-Swap model (Bays, 2016) hinges critically on the strong theoretical assumption that items in memory are independent of one another (in contrast to, e.g., Bae & Luck, 2017; Brady & Alvarez, 2011, 2015; Chen et al., 2019; Chunharas et al., 2019; Lively et al., 2021; Golomb, 2015; Scotti, Hong, Leber & Golomb, 2021). Overall, we argue that it is critical for working memory researchers interested in measuring swaps to motivate and test the particular theory underlying the measurement of swap rates rather than attempt to use any of the swap models as purely a measurement model for estimating swap rates.

## Simulation Methods

In this section, we report results from extensive parameter recovery simulations, which we used to evaluate the degree to which this set of models is robust across different theoretical and parametric assumptions. We underscore that the goal of our work is *not* to engage in model comparison; that is, our goal is not to promote a specific theory or model of visual working memory. A comparison between resource, mixture, and discrete-slot models is outside of the scope of this work and there is over a decade worth of literature that compares this suite of models and this topic is still actively debated (e.g., Ma et al., 2014; Luck & Vogel, 2013). Furthermore, work that challenges the “independence assumption,” which is made by both classes of models, is a newly, actively explored research area (e.g., Lively et al., 2021). Our work does not aim to provide a solution or comment on these fundamental questions. Instead, as discussed, our goal is to examine the degree to which contemporary swap models are robust across different processing assumptions by evaluating the degree to which swap rate estimates from each model are aligned with one another.

To this end, we simulated data from one swap model (the data-generating model) and evaluated the degree to which swap rate estimates from all three models are aligned with one another on this simulated data. That is, we address the following basic question: If we fit any of these three models to estimate swap rates, and all fit the data well, would we reach the same conclusions regarding how, e.g., a given experimental condition affects swap rates? As previewed in the introduction, the answer to this question is “no,” indicating that all three models rely on a specific set of theoretical assumptions that, if violated, would lead to incorrect estimates of swap rate.

For simplicity, we chose the TCC-Swap model as the generative model for the simulations reported in the main text. Note that, for the purpose of our question, it does not matter which model we choose as the generative model because, for our purposes, it is sufficient to demonstrate that there is a generative model, theory, and a set of processing assumptions that differentially affects swap rates for each model. The reason we chose the TCC-Swap model is because it has fewer parameters than the Mixture-Swap model and, in general, mixture models—like the Bays et al. (2009) model—can nearly perfectly “mimic” the TCC-Swap model (as shown by Schurgin et al., 2020). That is, the TCC-Swap model makes predictions—and generates simulated data—that fall into a smaller subset of the possible outcome space (Roberts & Pashler, 2000) and consequently, the Bays et al. (2009) model can nearly perfectly fit *any* possible error distribution generated by the TCC-Swap model. However, the converse does not hold—there are many combinations of the “guess rate” and “precision” parameters from the Mixture-Swap model that cannot be adequately fit by the TCC-Swap model (Schurgin et al., 2020; Williams et al., 2022). In the current context, it would be uninformative to report parameter recovery simulations for a model that fails to adequately fit the distribution of errors generated by an alternative model. Even if the two models yield similar swap rate estimates, this result would be difficult to interpret if the TCC-Swap model fails to adequately capture patterns in the data^1^ generated by Bays et al. (2009) swap model. Thus, for all of the simulations reported here, we use the TCC-Swap model as the generative model. However, to be systematic, we have also repeated these analyses by generating data from the Bays et al. (2009) model using combinations of parameters that TCC-Swap could mimic. These analyses resulted in identical conclusions.

In the following sections, we describe the technical details of each model, each of our simulations and findings. We also provide guidance to researchers by discussing the processing assumptions that lead to substantial divergences in swap rate estimates for these models.

### The TCC Model

The TCC model is a recent proposal for how continuous report memory distributions can be quantified (Schurgin et al., 2020). It argues that within a given stimulus space, memories are characterized by only a single parameter—memory strength (i.e., *d’*). In particular, the TCC model claims that for any given stimulus space (e.g., for any given color wheel), there is a fixed perceptual similarity function that quantifies how confusable items are, and this function is not linearly related to distance along the color wheel—rather, it is roughly exponential when averaged across all stimuli in the space (e.g., Nosofsky, 1992; Shepard, 1987) and can be measured in an independent task (see Schurgin et al., 2020).

Understanding this stimulus confusability function allows a simple signal-detection model to explain memory performance across a huge variety of conditions, with only a single parameter (*d’*). In particular, consider the case of a color working memory experiment. On any given trial, the to-be-remembered color is boosted by a strong familiarity signal (strength: *d’*), and completely dissimilar colors do not have their familiarity signal boosted at all. Intermediate colors have their familiarity signals boosted proportional to how similar they are to the target. So, a color 1° away from the to-be-remembered color gets a large boost in familiarity, and a color 10° away from the to-be-remembered color gets a moderate boost in familiarity. Noise is then added to these familiarity signals, and when participants are asked what color they saw, they report the color that has the highest familiarity.

Formally, this means the continuous report task is conceptualized as a 360-alternative forced choice task: Let *f(x)* be how similar a given color is to the to-be-remembered color. Let (*X*_-179_, …, *X*_180_) be a vector of normal random values with means *d*_*x*_ = *d’ f(x)* and unit variance. Then the reported color value, *r*, on this trial is simply:

$$r\sim \text{argmax}\left(X_{-179},\ldots ,X_{180}\right)$$

In the current simulation, we rely on the similarity data of Schurgin et al. (2020) and their technique for fitting the model, including the necessary correlation between colors based on perceptual matching data to adjust *d’* (Schurgin et al., 2020).

### The TCC-Swap Model

We use the TCC-Swap model proposed by Williams et al. (2022). In particular, we introduce a “swap” parameter into the TCC-Swap model to make a TCC-Swap model. Rather than assuming participants always report based on the similarity, *f(x)*, to the target item, we assume that on some trials, participants instead respond based on similarity to a non-target (i.e., the item at a non-tested location). We will take the simplest case, with just 2 items, one a target and one a non-target, as our case study. Let *f(x)* be the similarity to the target and *g(x)* be the similarity to the non-target. Let (*X*_−179_, …, *X*_180_) be a normal random vector with means *d*_*x*_ = *d’ f(x)*, unit variance, and let (*Y*_−179_, …, *Y*_180_) be a normal random vector with means *d*_*x*_ = *d’ g(x)*, and unit variance. Let (*β*) be the “swap rate.” Responses are generated as follows:

$$w\sim \text{Bernoulli}(\beta )\;r\sim w*\text{argmax}\left(Y_{-179},\ldots ,Y_{180}\right)+(1-w)\text{argmax}\left(Y_{-179},\ldots ,Y_{180}\right)$$

In other words, for each trial, participants report the maximum familiarity signal from either the target or non-target with some probability (*β*) of reporting from the non-target distribution.

Note that *d’* in this model is the memory strength of the items that are correctly reported: that is, it is only directly interpretable as an overall memory strength if we assume that “swap” trials do not have systematically weaker target item memories than non-swap trials (e.g., that “swaps” arise primarily from noise in location or another cue dimension, rather than from strategic decisions). Depending on your assumption about this, memory strength on average across trials can be interpreted as either *d’* (if we assume equal strength on swap trials) or all the way down to *d’* * (1-β), if we assume zero strength for the truly probed item on swap trials. For a further discussion of this issue, see Williams et al. (2022).

### The Mixture-Swap Model of Bays et al. (2009)

The Bays et al. (2009) model assumes responses are generated from 3 components: a guessing component (1/360 for each response); a target-based component, distributed as a vonMises (μ = target, SD), where SD is the standard deviation; and a non-target component, in this case, with a simulation of just one non-target, a vonMises ($\hat{\mu }=\text{ non-target}$,SD). The model combines these in a weighted average:

$$r\sim (1-\beta -g)\text{vonMises}(\mu ,\sigma )+(\beta )\text{vonMises}(\hat{\mu },\sigma )+(g)\frac{1}{360}$$

The standard deviation of responses (SD) and the frequency of guesses (g) and non-target responses (β) are free parameters.

### The Non-parametric Swap Model of Bays (2016)

The NP-Swap model of Bays (2016) postulates that response errors in the delayed estimation task can be captured by an unknown distribution *f* (*x*). They then suppose that response errors may be made with respect to the probed item (reflecting latent imprecision of memory for the probed item), or response errors may be made with respect to a non-probed item (reflecting swap errors). Accordingly, *f* (*x*) defines the probability of a mixture of errors made with respect to the probed item and non-probed items presented in the memory array. Formally, on trial *j* of *n* trials and *m* items, the probability of making a response error of some magnitude *r* given item values *φ* is given by the following formula:

$$p\left(r|\varphi_{j,i}\cdots \varphi_{j,m}\right)=\sum_{k=1}^{m}\alpha_{j,k}f\left(\varepsilon_{j,k}\right)$$

where $\sum_{k=1}^{m}\alpha_{j,k}=1$ and *ε*_*j*,*k*_ are observed deviations (in radians) of responses on trial *j* with respect to item *k*. *α*_*k*_ captures the expected proportion of times that responses are made with respect to item *k* in the memory array. For example, in a color memory task, assume that *k* = 1 is the index for the target color and *k* > 1 are indices for distractor colors. If there are no swap errors, then *f*(*x*) defines the probability of errors made with respect to the target color only and *α*_*k*=1_ = 1 and *α*_*k*≠1_ = 0. In contrast, if there are swap errors, then the proportion of swap errors is equal to 1 − *α*_*k*=1_ where *α*_*k*≠1_ > 0.

Through the lens of this model, *α*_*k*_ is defined as the ratio of circular mean errors—with respect to each item the memory array—to the sum of circular mean errors relative to all items in the memory array. Conceptually, this follows from the fact that all errors are generated from the same distribution *f*(*x*), and *α*_*k*_ captures the proportion of times that errors from *f*(*x*) are made with respect to each item in the memory array. Formally, the circular mean for each item in the array across all trials is given by formula below, where *i* is an imaginary number:

$$\mu_{k}=\sum_{j=1}^{n}e^{i\varepsilon_{j,k}}.$$

Accordingly, the circular mean for all *m* items is $\mu_{T}=\sum_{k=1}^{m}\mu_{k}.$ Finally, *α*_*k*_ is obtained as the real part of the ratio of the mean error of each item with respect to the total mean error for all items, and is given by the following formula:

$$\alpha_{k}=Re\left(\frac{\mu_{k}}{\mu_{T}}\right).$$

The real part of this ratio is equivalent to the ratio with respect to the direction of *μ*_*T*_, and is used because it is a more reliable estimator, as reported in Bays (2016). It is important to note that because the NP-Swap model does not provide a distribution of responses, it cannot be plotted on top of an error histogram (as in Figs. 2 and 3). It is simply a metric to estimate swap rates.

### Simulation 1

We simulated 1000 subjects, each with 500 trials of memory responses, from the TCC-Swap model at various memory strengths (*d’* 0.5 to 4 in increments of 0.5). For each set of 500 trials, most responses were based on the target item, but some proportion of responses was based on a single non-target item that was randomly chosen. (We focus on a single non-target for simplicity but the same divergences arise in the same way with more non-targets.)

We simulated conditions where 20% of responses were based on the non-target and where 50% were based on the non-target. In all cases, we fit the TCC-Swap model to the data, the NP-Swap model, and the Bays et al. (2009) model as implemented in MemToolbox (Suchow et al. 2013a, b). The fits to the data of the two parametric models were excellent (see Figs. 2 and 3), since, as noted above, the Mixture-Swap model can nearly perfectly mimic the TCC-Swap model (see Schurgin et al., 2020). Our main interest was the resulting parameter estimates, and in particular the extent to which “swap” estimates were similar or distinct between the two models.

### Simulation 2

To demonstrate how the models deal with non-independence between items (like attraction and repulsion), we ran a simulation with moderate amounts of attraction/repulsion between the two items but no genuine swaps. We set *d’* = 2 and sampled data from TCC. We choose random locations for the distractors relative to the target, a prerequisite of the NP-Swap model. However, when the target and distractor were within 90° of each other on the stimulus wheel, we introduced non-independence: the memory for the target was either systematically attracted toward or repelled from the distractor item (similar to findings from Chunharas et al., 2019; Lively et al., 2021).

### Simulation 3

Simulation 3 was again at a fixed memory strength. We set *d’* = 2 and sampled data from TCC-Swap. However, in this case, we did not include any attraction or repulsion between items. Instead, we added a constraint that is very commonly used in the literature when choosing items to show in a display: a minimum distance between each item, such that the distractor could not be within *X* degrees of the stimulus space to the target (as in, e.g., Schurgin et al., 2020), where in our simulations we varied *X* across the range of 0–45°. This is commonly done to reduce the ability of participants to group nearly identical colors together (e.g., Morey et al. 2015; Brady & Alvarez, 2015).

### Simulation 4

Nearly all experiments rely on comparisons between conditions that differ in some way other than solely swap rate (e.g., memory strength will vary as a function of set size or encoding time, as in the original Bays et al., 2009 paper). Implicit in the previous simulations is that, if the models differ in their estimate of swap rate across the manipulations we simulate (e.g., memory strength; attraction/repulsion), then this will artifactually create patterns across conditions if there is a mismatch between the real data-generating model and the model that is fit. That is, if two conditions differ in memory strength, for example—as nearly all conditions do—then the swap rate estimates of the different models will also differ. Thus, in Simulation 4, we draw attention to this by focusing specifically on what the models would find if the TCC-generated swap rate was identical across two conditions that happened to differ in memory strength. Here, we use an example experiment where delay varies as the critical manipulation. We take a memory strength of *d*’ = 2 in the simulated “short delay” and of *d*’ = 1 in the “long delay,” and a fixed swap rate of 0.20 at both delays. We simulated 100 sets of 20 subjects each with 100 trials each of long and short delay per subject.

## Results

**Fig. 1:**
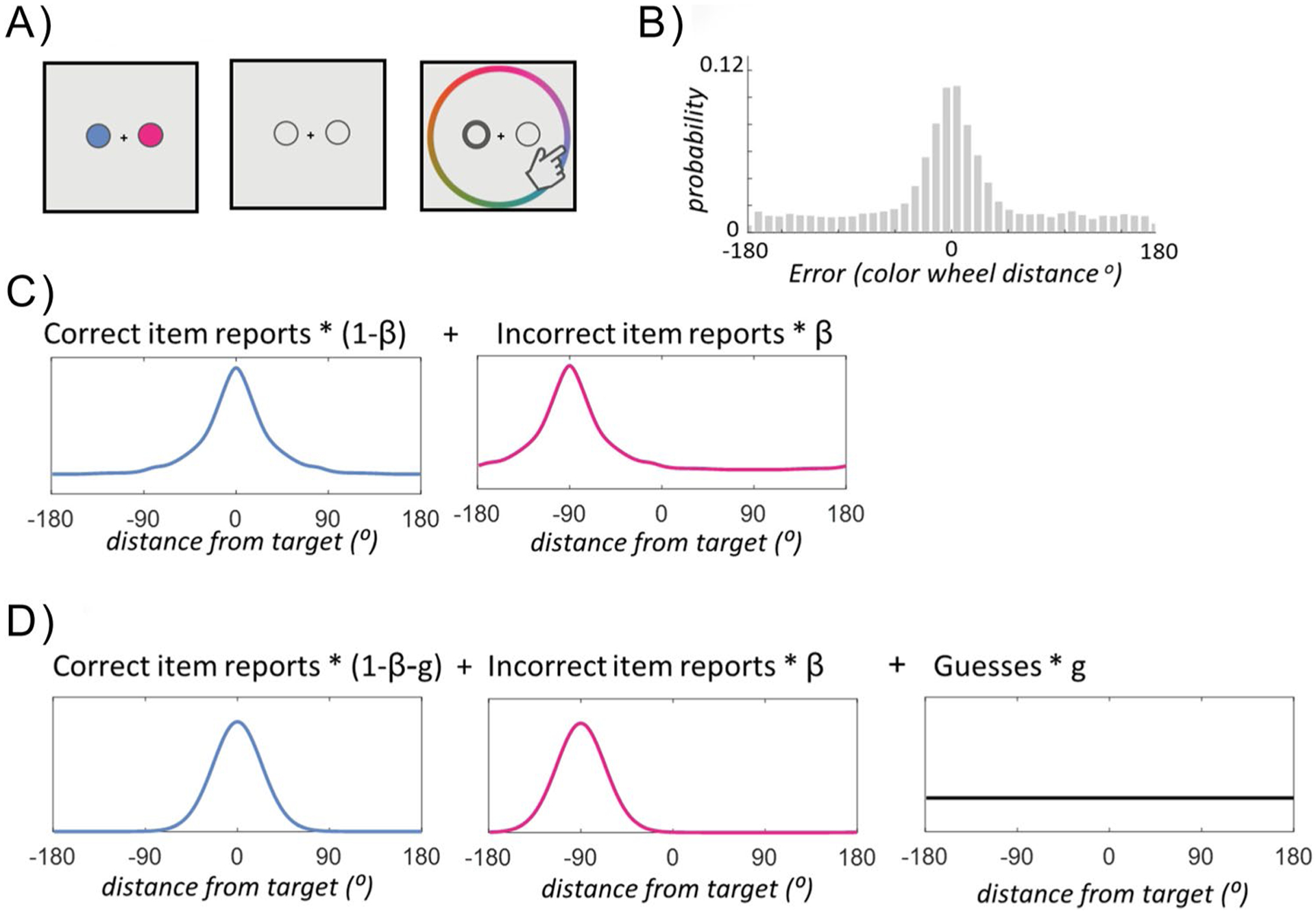
Contrasting swap models. **A** Imagine a color report task with just 2 items, but where performance is nonetheless made challenging by increasing delay, decreasing encoding time, or some other manipulation. **B** A typical pattern of data in such a task, aggregated across trials, shows many small errors—near 0, which is the exactly correct color—but also many large errors (the long tail to the left and right, of reports far from the correct answer). Different models have competing views of why this long tail arises (e.g., guessing vs. noisy item-based responses). If swaps are present, they are usually masked in such aggregated data by the fact that, on average across trials, the non-probed item (the non-target) is randomly related to the probed item (the target). **C** The TCC model (Schurgin et al., 2020), along with other influential models of working memory (e.g., Schneegans et al. 2020; van den Berg et al., 2012), proposes that even individual item memories necessarily have “long tails” (e.g., large errors, due to noise). In such a model, when adding in “swaps,” these swaps also have such long tails. That is, if you mistakenly respond based on an incorrect item, you will still sometimes make large errors in response. **D** By contrast, typical mixture model instantiations of swaps, based on Bays et al., 2009’s influential model, suppose that item reports themselves do not have a long tail, but that this long tail of errors instead arises from guessing. Thus, while both can account for similar error distributions, they end up with fundamentally different interpretations of these distributions (e.g., in terms of how many swaps are present)

**Fig. 2:**
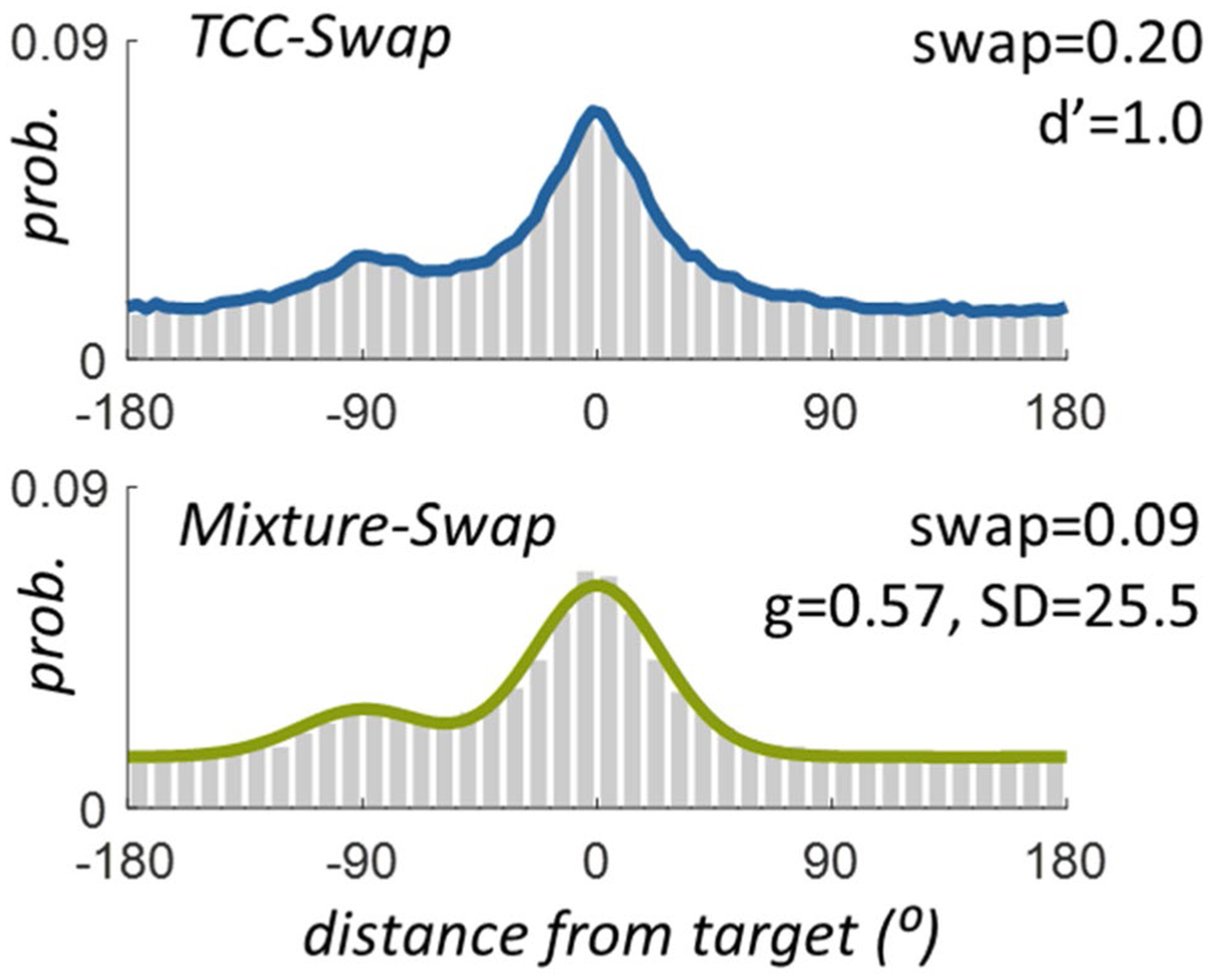
Differing interpretations. The differing interpretations between the models of what generates the long tail of errors (see Fig. 1) also necessarily result in different estimates of swap rates. Consider this simulated data, sampled from TCC-Swap with *d*’ = 1.0 and swap rate = 20% with a single non-target that in this case is reliably located, always at exactly − 90° from the target, so that averaging across trials does not hide the swap errors as it does in typical data. The two models can both fit the data very well, as it is generated from the TCC-Swap model and the Mixture-Swap model can mimic this model perfectly in terms of fit. However, despite both fitting the data, they arrive at very different estimates of swap prevalence: swap rates of 20% vs. swap rates of 9%. This is because the TCC model interprets part of the long tail as arising from swaps, whereas the Mixture-Swap model does not, allocating it entirely to “guesses”

**Fig. 3:**
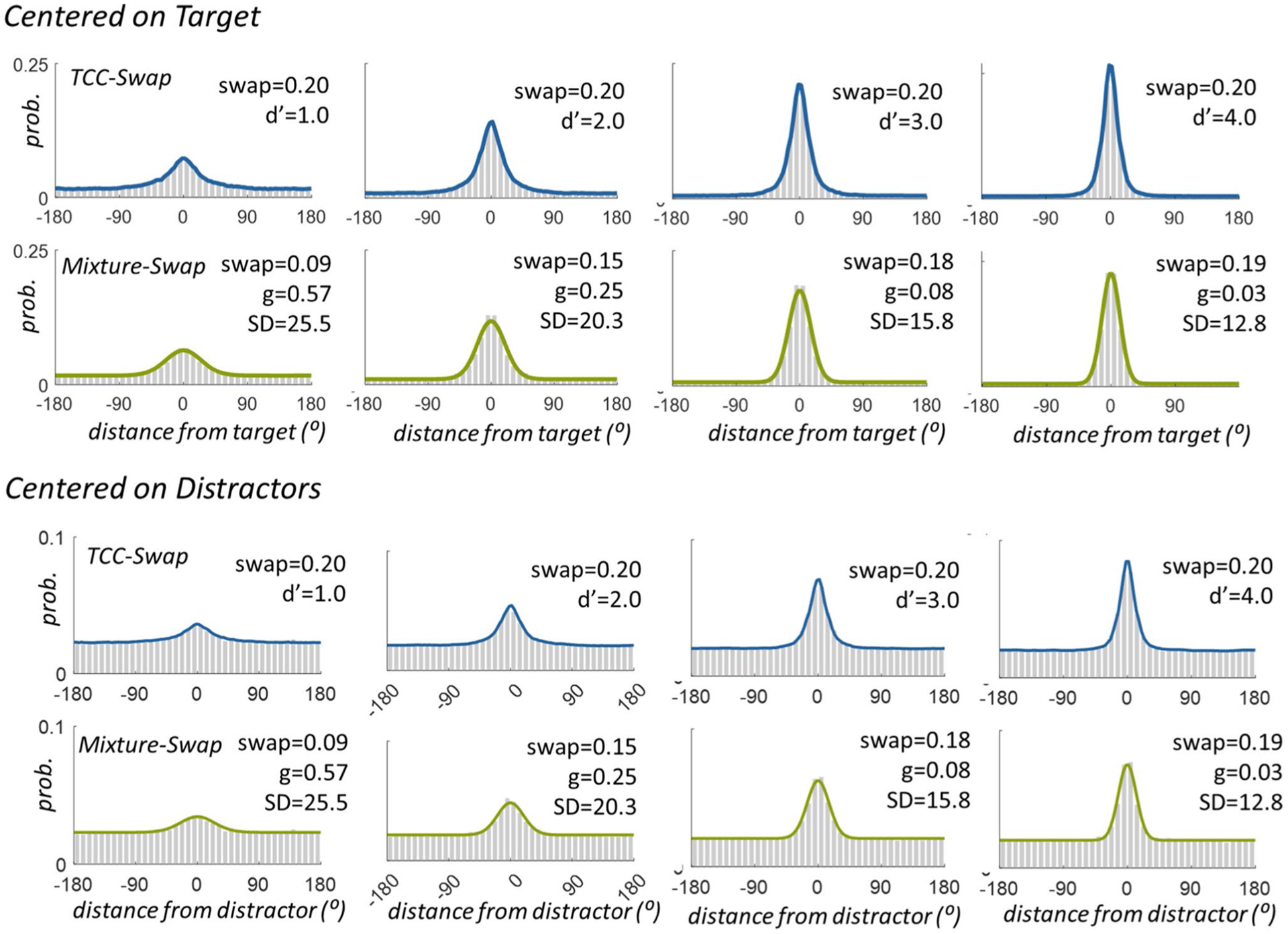
Results of fitting TCC-Swap and Mixture-Swap model to data sampled from TCC-Swap *d*’ = 1,2,3,4 with swap rate 0.2. The data is well fit by both models (as the Mixture-Swap model can mimic the TCC-Swap model nearly perfectly), but results in quite different swap rates. (The non-parametric model of Bays (2016) does not provide a distribution of responses and so cannot be shown; it just a metric to estimate swap rates)

**Fig. 4:**
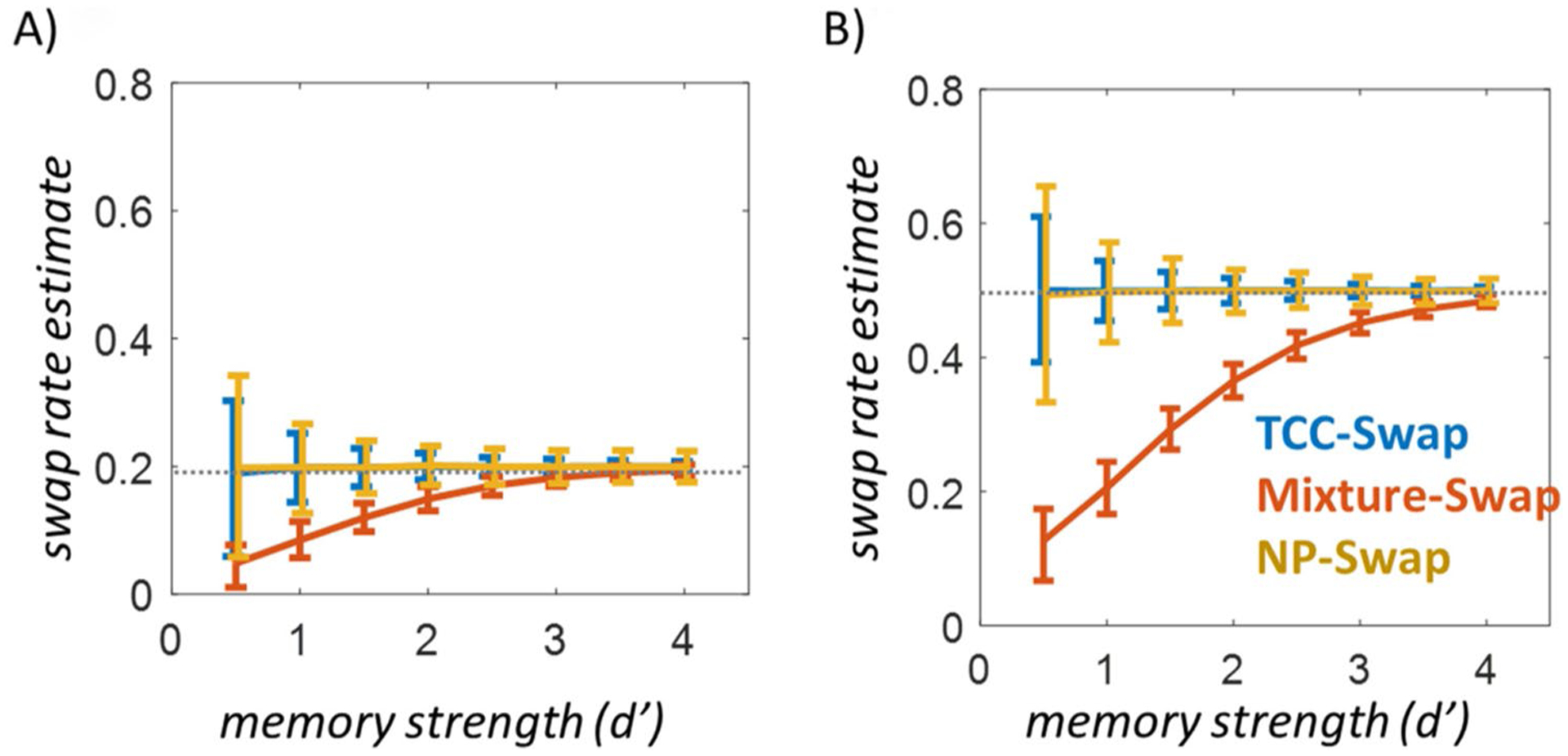
Simulation 1. Simulating 500 trials per subject from the TCC-Swap model at each of various memory strengths and plotting mean recovered swap parameters (with standard deviation across subjects plotted as error bars). In **A** simulation, the true “swap” rate is 20%; that is, 20% of responses are generated based on the non-target item, according to the TCC-Swap model. As *d*’ drops, the Mixture-Swap model assigns responses to the “guess” distribution that, in TCC, are generated from the non-target distribution (as there are no guesses in the TCC-simulated data). *d*’ values below 2 are typical at high set sizes where swaps tend to arise (Schurgin et al., 2020), so the deviation between the models is critical to the interpretation of the vast majority of papers that fit swap models. Because the NP-Swap model assumes that all responses come from an unknown shape of item-based response distribution—with no separate conception of guessing—it arrives at the same conclusion as the TCC-Swap model, with less certainty in the estimate. **B** Similar results arise when generated from data with 50% swaps, with the deviations between Bays et al. (2009) and TCC-Swap/NP-Swap model becoming more important earlier (i.e., at higher memory strengths)

The Mixture-Swap model dissociates strongly from the TCC-Swap model and the NP-Swap model in Simulation 1 (Fig. 4). The Mixture-Swap model (Bays et al., 2009) arrives at systematically different conclusions than the other two models primarily when memory is weak (Fig. 3A and B), but always agrees when memory is strong. This would be expected since the interpretation of the long tail of errors is the critical distinction between these models, and this long tail is strongest and thus has the greatest effect on estimates of swap rates when memory is weak. Both the TCC-Swap and NP-Swap models make the theoretical assumption that swap responses have long tails, whereas the Mixture-Swap model assumes the opposite (see Fig. 1D). Thus, these models do not reliably agree even in incredibly straightforward simulated data with only a single distractor present—in fact, they produce qualitatively distinct conclusions as memory strength and swap rate vary.

**Fig. 5:**
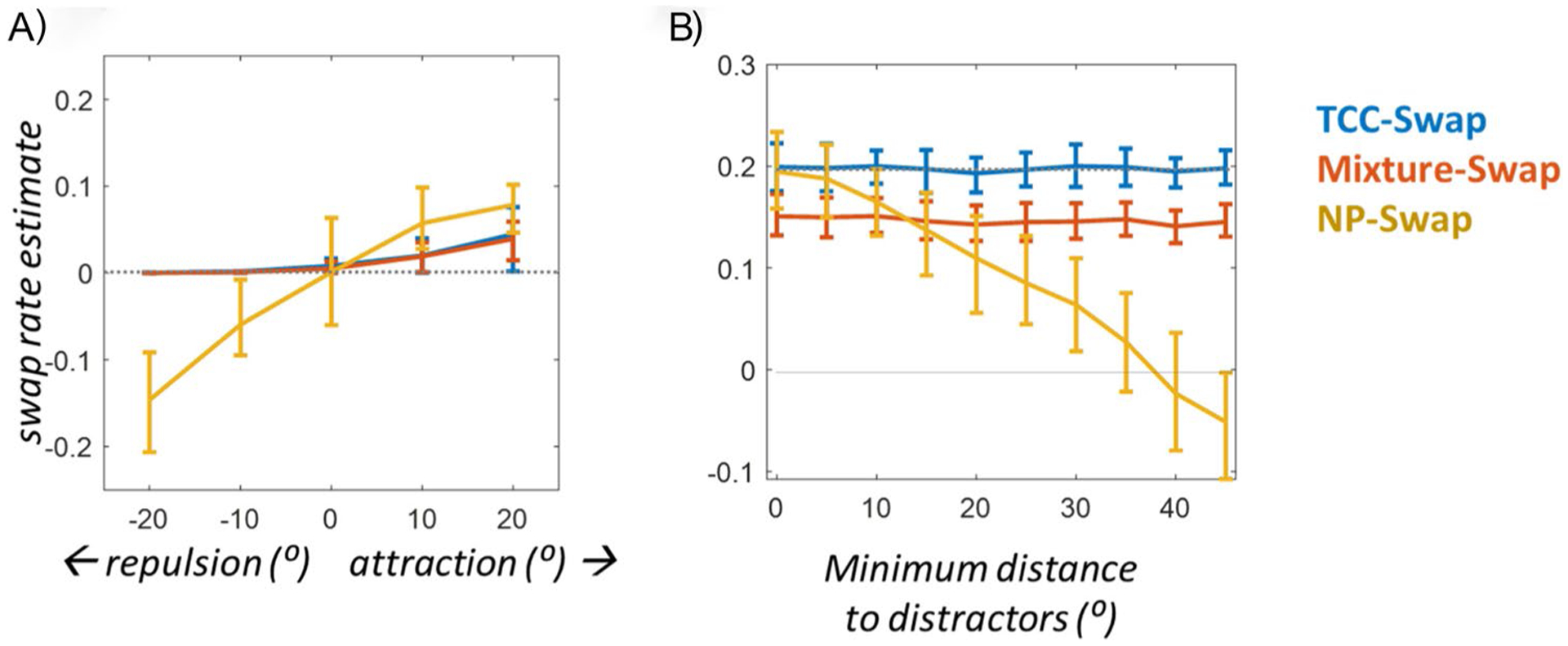
Simulations 2 and 3 demonstrate the necessary requirements for valid inference from the Bays (2016) model. **A** Results of swap model fits in Simulation 2, where the 2 items repel or attract each other when within 90° on the color wheel, but there are no genuine swap errors present. When there is no attraction or repulsion (0 on the x-axis), all models correctly recognize the 0 swap rate, though the Bays (2016) model has considerable uncertainty compared to the other models. However, when attraction or repulsion is present, the Bays (2016) model reliably confuses attraction and repulsion with swapping—and negative swapping—which means that when genuine swaps are present, any repulsion that is also present will result in a significant underestimate of swap rate in this model. This occurs because this model has no knowledge of the parametric form of the item-based responses (e.g., that it should, in the absence of biases, be centered directly on the item), and so it cannot separate a shift in the distribution from a change in the shape of the distribution. **B** Simulation 3 shows what happens when fitting the models to data where the distractors are required to be a certain minimum distance from the target (and usually, each other). This is a commonly used technique to reduce grouping and ensemble usage, but, as noted by Bays (2016), causes the non-parametric model to underestimate swapping and even end up with negative swap estimates

Simulations 2 and 3 focus on how violations of assumptions of the NP-Swap model that commonly occur in real experiments affect its ability to recover true swap rates (Fig. 5). In particular, these simulations highlight how if certain fundamental and strong assumptions are not met, the NP-Swap model confounds inter-item distortions with “swaps.” As seen in Fig. 4A, Simulation 2 shows that, even with no swaps present, the NP-Swap model yields estimates of both positive swaps (when items attract) and negative swaps (when items repel each other). This occurs even when, like in these simulated data, the distractors are randomly distributed across trials and there is constraint on how close they are to the target item in feature space (a prerequisite for this model). These results are especially problematic because extensive evidence suggests that attraction and repulsion between items are nearly ubiquitous (e.g., Bae & Luck, 2017; Brady & Alvarez, 2011, 2015; Chen et al., 2019; Chunharas et al., 2019; Lively et al., 2021; Golomb, 2015; Scotti, Hong, Leber & Golomb, 2021) and that variables like memory strength, encoding time and delay—which change swap rates (e.g., Bays et al., 2009)—also change attraction and repulsion between items systematically (Chunharas et al., 2019; Lively et al., 2021).

By contrast, Simulation 3 elucidates how the NP-Swap model also yields inaccurate swap rate estimates if there are no memory biases but, as is common practice, distractors and the target are required by the experimenter to be a minimum distance in stimulus space from each other (e.g., no items may be closer than 15 deg, as in Schurgin et al., 2020). Although this constraint is commonly used in visual working memory tasks to reduce swap rates, it too violates the assumptions of the NP-Swap model and causes it to underestimate swap rates considerably. While this was noted by Bays (2016), the effects of this were not visualized in that work. We find they are quite substantial even at relatively small minimum distances between the shown items.

Since nearly all experiments rely on comparisons between conditions, we focused on a comparison between two conditions where memory strength, but not swap rate, changed in Simulation 4. Implicit in the previous simulations is that, since the models differ in their estimate of swap rate across the manipulations we simulate (e.g., memory strength; attraction/repulsion), then this will artifactually create patterns across conditions if there is a mismatch between the real data-generating model and the model that is fit. That is, if two conditions differ in memory strength, for example—as nearly all experimental conditions do—then the swap rate estimates of the different models will also differ. Thus, in Simulation 4, we compared the models using data where the TCC-generated swap rate was identical across two conditions that happened to differ in memory strength (which is something one might find if they varied delay across conditions, for example). We use a memory strength of *d*’ = 2 in the simulated “short delay” and of *d*’ = 1 in the “long delay,” and a fixed swap rate of 0.20 at both delays. As would be expected, both the TCC-Swap and NP-Swap models recover swap rates of nearly exactly 20% in both conditions, and on average find no difference between the conditions in swap rates (*p* < 0.05 in 2% of simulations for TCC-Swap and 6% of simulations for the NP-Swap model, consistent with the expected alpha level of the *t* test). By contrast, the Mixture-Swap model is estimated on average swap rates of 15% in the *d*’ = 2 condition and 9.8% in the *d*’ = 1 condition, which was statistically different (*p* < 0.05) in 79% of simulations. Thus, using the Mixture-Swap model would suggest a decrease in swap rates with delay, whereas using the other two models would not. A similar situation would naturally arise if data was generated from the Mixture-Swap model with a fixed swap rate: as memory strength decreased, the TCC-Swap and NP-Swap models would estimate that swap rates increased, even though in the Mixture-Swap model, they would be fixed.

## General Discussion

In a series of simulations, we evaluated three swap models: one based on the common “Mixture-Swap” model of Bays et al. (2009), one based on the TCC model of Schurgin et al. (2020), and a third non-parametric swap model that is assumed to rest on minimal theoretical assumptions (Bays, 2016). First, we find a stark disagreement between the Mixture-Swap and TCC-Swap models in estimating swap rate, particularly when memory is weak. This discrepancy arises because of different interpretations of the “long tail” of errors that arise in such tasks. Overall, the TCC-Swap model estimates much higher swap rates than the Mixture-Swap model and does so differentially when memory is weak.

It has generally been found that swaps arise primarily when items are presented for brief durations and when many items are presented (e.g., Bays et al., 2009; Emrich & Ferber, 2012), factors that also reduce memory strength more generally. Thus, the large discrepancy in swap estimates across these models raises a significant conceptual hurdle for interpreting the effects of such manipulations on swap rates, and visual working processes more generally. The increasing prevalence of models that does not postulate complete information loss in visual working memory, or distinct “guess state” (Bays, 2015; Schurgin et al., 2020; Schneegans et al. 2020; van den Berg et al., 2012), underscores the need to take seriously the idea, instantiated in the TCC-Swap model in this particular set of simulations, that swaps may be more prevalent when measured at low performance levels than previously thought. At a minimum, studies relying primarily on swap measures should carefully consider the implications of these simulations and the possibility of distinct interpretations of their results.

These results have major implications for nearly all measurements of swapping in visual working memory, dating back to the very first claims of Bays et al. (2009). As shown in Simulation 4, comparing swap rates across conditions that vary in memory strength is nearly entirely dependent on whether the long tail of errors is considered to be item-based (and thus contain swaps) or based on guessing (and thus not contain swaps). Thus, comparisons of swap rates between conditions that differ in memory strength are particularly difficult to make. Consider the original finding of Bays et al. (2009) that encoding time affected the guess rate but not the swap rate, which they interpret as showing that short encoding time leads to more random responding but does not affect the encoding of the items’ location. This finding would be different if analyzed using a more modern model that did not include guesses, like Bays (2016) or the TCC-Swap model, as the increase in “guess” responses would also mean an increase in “swap” responses (since some of the long tail of responses would be associated with swaps, as in Simulation 1). In general, any claim that swap rates are similar when memory strength differs or vice versa is extremely dependent on the particular swap model that was used, and many experiments make such claims. To take a few examples (of many possible), Borders et al. (2022) compare hippocampal lesion patients with controls and find similar swap rates using Mixture-Swap despite an overall weaker memory strength among patients, and take this as evidence that hippocampal lesions do not impact swaps; with a swap model that includes swaps in the “tail” of the distribution, the weaker memory strength of patients would likely also include additional swaps. Similarly, Zokaei et al. (2020) compare Alzheimer’s patients, Parkinson’s patients, and subjective cognitive impairments and find different patterns of memory strength vs. mis-binding errors in the different groups, but they do so using a Mixture-Swap model, and make several comparisons that compare swap rates across groups with different memory strengths, etc. Overall, such comparisons are common in the literature, and as highlighted in Simulation 4, lead to strongly different conclusions depending on the way the long tail of errors is treated when estimating swaps.

Our results also show that researchers should also carefully check for the presence of attraction and repulsion effects between items when using the NP-Swap model (Bays, 2016), which we found strongly confounds these effects with “swap” errors. Although not shown in the current simulations, in previous work, we have shown that other swap models also confound conceptually similar effects, including “ensemble effects,” with “swaps.” For example, Utochkin and Brady (2020) showed that the Mixture-Swap model estimates higher “swap” rates when the range of the display is smaller (e.g., when all the items are within 90° on the stimulus wheel), even though there is no reason to believe swap rates would be different for these kinds of displays. Instead, this is almost certainly spurious, arising because participants tend to limit their responses only to reasonable values that could have been present based on their knowledge of the entire display, and this results in many responses near non-targets when the items are all clustered together. A similar point, framed in terms of high threshold “guessing,” was made by Pratte (2019), who showed that people tend to use knowledge of the other items in the display to limit their responses when they have low confidence, and these strategies can be captured as “swaps” by “swap” models.

Our work emphasizes that measuring a particular amount of “swaps” does not provide an explanation of their generative processes. For example, swaps may occur because of location confusions, and thus be relatively orthogonal to memory strength for the items themselves (e.g., McMaster et al., 2022; Oberauer & Lin, 2017). However, they may also reflect strategic uses of ensemble or other item information when memory is weak (e.g., Pratte, 2019). Therefore, a measurement of swap rates does not yield an unambiguous measure of how items are represented in visual working memory, nor even the true rate at which responses were “generated” from reporting the memory for an incorrect item. In addition to the ambiguity in interpreting swap rates themselves, which we show hinges on the commitment to a particular model of memory errors, the same is true for interpreting true levels of “memory strength” in the presence of swaps. For example, consider the TCC-Swap model. Due to the parsimonious nature of the TCC framework, if this theory is embraced, estimating memory strength in the presence of swap rates is relatively straightforward, but still ambiguous. This is because TCC postulates that memory strength lies on a single continuum, which is quantified with the signal-detection theoretic parameter *d’*. Accordingly, the TCC-Swap model is committed to the idea that if swap trials have the exact same latent memory strength as non-swaps, then *d’* is the best estimate of overall memory strength; but if swap trials result when people are purely guessing— e.g., arise when *d*’ for the target was 0, then target memory strength is (*d*′(1 – *β*)). Therefore, it is in principle possible to recover an upper bound (*d*′) and lower bound (*d*′(1 – *β*)) for memory strength in a given experimental condition even in the presence of swap rates (Williams et al., 2022).

One final factor, not discussed in this work—as it is common across all the models—is that a shared assumption of each of these models is that swaps and responses to the probed item are generated from the same latent distribution. It follows that all models share the potentially untenable assumption that reports on probed and non-probed items share the same latent memory strength. If trials where people make a swap error are not randomly related to the strength of memory (e.g., if people are more likely to report an incorrect item on trials where they had a weak item memory), all of these models will give incorrect estimates of swap rates. It seems likely that this must be true to some extent: e.g., if swaps in a color report task cued by location arise from noisy location memory (e.g., McMaster et al., 2022; Oberauer & Lin, 2017), then any correlation between the strength of color memory and location memory across trials—for example, fatigue or attentional factors—would violate this assumption. Testing this in real data is difficult, and it is not a factor that distinguishes the current set of models. However, it is a critical aspect of all of these models to consider when interpreting swap rates from them.

Another factor not addressed in the current simulations is that the parametric form of the distributions assumed for non-swap responses are different between different models (e.g., the TCC model, like the van den Berg et al. (2012) variable precision model, is “peakier” than the Bays et al. (2009) and Zhang and Luck (2008) mixture model; see Schurgin et al., 2020). In addition to the implications of this for fitting data without swaps that have already been well-discussed in the literature, there are also implications of this when fitting swap models, particularly when the distractor locations are fixed across trials. For example, if distractors are always 60° away from the target, the fact that the models predict—without any swaps included—different shape error distributions at that point means there may be a bias in the fit of the swap models as well. For example, to account for the data being “peakier” than the model, swaps might be estimated as higher than they truly are in the Bays et al. (2009) model if distractors tend on average to be close to the target; or may end up underestimated in this model if distractors are reliably far from the target. In general, all of the models will have an interaction between how well they fit the non-swap responses and how they account for swaps.

Overall, we make the following recommendations. First, researchers should be aware that no extant measurement model of swap rates is assumption-free. The point that measurement and theory mutually constrain each other is not specific to estimates of swap rates. Measurement of unobservable processes cannot be done without a guiding theoretical framework (e.g., Brady et al., 2021; Kellen et al., 2020), which rests on specific core theoretical assumptions about latent processes (e.g., item versus resource based limits in visual working memory), as well as its own set of auxiliary and simplifying assumptions. All of these assumptions can affect the interpretation of the construct being measured in ways that are specific to that theory. In line with this, our simulations demonstrate that such assumptions can, in the case of swap models, profoundly affect the estimates of swap rates and lead to discrepant quantitative and qualitative conclusions about visual working memory processing. Thus, researchers should (1) check that their results and conclusions are robust across different swap models, and if they are not, (2) motivate their choice of a swap model in a principled fashion. To this point, most contemporary models of visual working memory suppose that even very large, long-tailed errors can arise from item-based representations, rather than pure guessing (Schurgin et al., 2020; Schneegans et al. 2020; Bays, 2015; van den Berg et al., 2012); therefore, we would warn against an unprincipled use of the Mixture-Swap model (Bays et al., 2009). We also promote the development of non-parametric models, like the NP-Swap model of Bays (2016), that are more robust to known violations of independence between items, like attraction and repulsion (e.g., Chunharas et al., 2019; Lively et al., 2021) and other significant and complex interactions across items in a display that are commonly observed (Brady & Alvarez, 2015; Oberauer & Lin, 2017; Swan & Wyble, 2014).

Together, our work fits with the broader idea that “measurement models” cannot be used independently of the underlying theory or parametric assumptions of how memory responses are generated. Our simulations are the first to demonstrate how this point applies in the estimates and interpretation of swap rates and visual working memory processing more generally. We hope that our work serves as a cautionary note as well as a guide for researchers invested in understanding the architecture of this fundamental memory system.

## Data Availability

See “Code Availability.”

## Footnotes

1 Note that this does not imply that the TCC model accommodates real working memory data worse that the Bays et al. (2009) model. Rather it means that the TCC model is comparatively less flexible and cannot mimic large amounts of the *possible* data that can be generated by the Bays et al. (2009) model. Importantly, real data seem to come from the subset that the TCC model predicts (see Schurgin et al., 2020).
